# Immune-Inflammatory Parameters in COVID-19 Cases: A Systematic Review and Meta-Analysis

**DOI:** 10.3389/fmed.2020.00301

**Published:** 2020-06-09

**Authors:** Xudong Feng, Shuangshuang Li, Qiang Sun, Jiaqi Zhu, Bo Chen, Maoming Xiong, Guodong Cao

**Affiliations:** ^1^State Key Laboratory for the Diagnosis and Treatment of Infectious Diseases, College of Medicine, The First Affiliated Hospital, Zhejiang University, Hangzhou, China; ^2^Department of Microbiology and Center of Infectious Disease, School of Basic Medical Sciences, Peking University Health Science Center, Beijing, China; ^3^Jiangsu Key Laboratory of Biological Cancer, Cancer Institute, Xuzhou Medical University, Xuzhou, China; ^4^Department of General Surgery, The First Affiliated Hospital of Anhui Medical University, Hefei, China

**Keywords:** COVID-19, immune-inflammatory parameters, neutrophil-lymphocyte ratio, risk factor, meta-analysis

## Abstract

**Background:** The recent outbreak of coronavirus disease 2019 (COVID-19) has been rapidly spreading on a global scale and poses a great threat to human health. Acute respiratory distress syndrome, characterized by a rapid onset of generalized inflammation, is the leading cause of mortality in patients with COVID-19. We thus aimed to explore the effect of risk factors on the severity of the disease, focusing on immune-inflammatory parameters, which represent the immune status of patients.

**Methods:** A comprehensive systematic search for relevant studies published up to April 2020 was performed by using the PubMed, Web of Science, EMBASE, and China National Knowledge Internet (CNKI) databases. After extracting all available data of immune-inflammatory indicators, we statistically analyzed the risk factors of severe and non-severe COVID-19 patients with a meta-analysis.

**Results:** A total of 4,911 patients from 29 studies were included in the final meta-analysis. The results demonstrated that severe patients tend to present with increased white blood cell (WBC) and neutrophil counts, neutrophil-lymphocyte ratio (NLR), procalcitonin (PCT), C-reaction protein (CRP), erythrocyte sedimentation rate (ESR), and Interleukin-6 (IL-6) and a decreased number of total lymphocyte and lymphocyte subtypes, such as CD4+ T lymphocyte and CD8+ T lymphocyte, compared to the non-severe patients. In addition, the WBC count>10 × 10^9^/L, lymphocyte count<1 × 10^9^/L, PCT>0.5 ng/mL, and CRP>10 mg/L were risk factors for disease progression in patients with COVID-19 (WBC count>10 × 10^9^/L: OR = 2.92, 95% CI: 1.96–4.35; lymphocyte count<1 × 10^9^/L: OR = 4.97, 95% CI: 3.53–6.99; PCT>0.5 ng/mL: OR = 6.33, 95% CI: 3.97–10.10; CRP>10 mg/L: OR = 3.51, 95% CI: 2.38–5.16). Furthermore, we found that NLR, as a novel marker of systemic inflammatory response, can also help predict clinical severity in patients with COVID-19 (OR = 2.50, 95% CI: 2.04–3.06).

**Conclusions:** Immune-inflammatory parameters, such as WBC, lymphocyte, PCT, CRP, and NLR, could imply the progression of COVID-19. NLR has taken both the levels of neutrophil and lymphocyte into account, indicating a more complete, accurate, and reliable inspection efficiency; surveillance of NLR may help clinicians identify high-risk COVID-19 patients at an early stage.

## Introduction

In late 2019, a novel corona virus (SARS-CoV-2) caused an outbreak of a cluster of pneumonia cases in Wuhan, China. The new 2019 coronavirus pneumonia, COVID-19, has continued to spread rapidly around the world and has since become a global health emergency. Although control and quarantine measures have been applied to prevent a global spread, the infection has gradually increased, resulting in a pandemic (1). As of April 22, 2020, a total of 2,528,330 COVID-19 cases and 177,198 fatal cases were reported worldwide, with 84,287 cases and 4,642 deaths reported in China alone. Moreover, the number of people infected with COVID-19 in the United States accounts for about one-third of the world, with a 5.5% mortality rate.

SARS-COV-2 is a member of the coronavirus family along with SARS-CoV and MERS-CoV, but its transmission speed and infectivity are stronger than both (2, 3). SARS-CoV-2 can be transmitted through the respiratory tract, mainly causing respiratory infections and developing severe pneumonia, respiratory failure, and even death in infected patients (4, 5). Although the current situation is very grim, there is no specific medicine available for SARS-CoV-2. A recent systematic review and meta-analysis reported that the COVID-19-related mortality rate varies widely among epicenters and counties, even at the global level (6). In addition, researchers have identified several clinical characteristics associated with an increased risk of developing severe COVID-19, such as hypertension, cardiovascular disease, chronic kidney disease, and diabetes (6). Considering that infections might progress rapidly in some patients and timely clinical decisions are required, identifying patients who are at risk of developing a serious disease is particularly important for healthcare workers.

Since the pathophysiology of unusually high pathogenicity for SARS-CoV-2 has not been completely explained, information about inflammation and the immune response of patients with different severity of COVID-19 remains insufficient. Inflammation accompanied by an immune response often occurs in viral respiratory infections, and increasing evidence supports its important role in the progression of COVID-19 (7). Moreover, a previous retrospective study reported that a subgroup of patients with severe COVID-19 could have a dysregulation of the immune response that allows the development of viral hyperinflammation (8). In view of the fact that inflammation or immune indicators are very common and easily obtained, identifying risk factors in blood associated with disease severity among SARS-CoV-2-infected patients is vital for early intervention to improve mortality.

However, to date, no systematic review has been reported concerning the putative association between various inflammation indicators and the progression of COVID-19. Therefore, in the current study, a systematic review and meta-analysis was conducted to summarize the difference of several inflammation indicators between severe and non-severe COVID-19 patients and identify the relevant risk factors correlated with the progression of COVID-19.

## Materials and Methods

### Literature Search Strategy

The PubMed, Web of Science, EMBASE, and China National Knowledge Internet (CNKI) databases were searched for eligible publications from December 2019 to April 2020. The search strategy was based on combination of following terms: “COVID-19” OR “Novel Coronavirus-Infected Pneumonia” OR “2019 novel coronavirus” OR “NCP” OR “2019-nCoV” OR “SARS-CoV-2.” The search items in each database are also available in Supplementary Material. References cited in the retrieved articles were also scanned for relevant studies. Two reviewers independently screened the title and abstract of each study. Articles deemed potentially eligible were retrieved for a full-text review.

### Selection and Exclusion Criteria

This systematic review and meta-analysis were conducted according to the PRISMA guidelines. Studies included in the meta-analysis had to satisfy the following criteria: (1) the study was a clinical observation in humans; (2) the study included clinical signs of a COVID-19 patient; and (3) the study included baseline information regarding inflammation indicators, such as white blood cells, neutrophils, lymphocytes, C-reactive protein (CRP), and procalcitonin (PCT). All articles of any design (randomized controlled trials, non-randomized controlled trials, case-control studies, and cross-sectional studies) were included except for narrative review, comment, opinion piece, methodological report, or conference abstract.

### Data Extraction and Quality Assessment

Data were extracted separately by two reviewers, and discrepancies were resolved by consensus. The following data of each study were extracted from included articles: name of first author, publication date, study location, sample size, patients' age, gender, study design, COVID-19 severity, and inflammation indicators. The quality of the included studies was assessed using the MINORS (9) by two independent reviewers. MINORS is a valid instrument designed to assess the methodological quality of non-randomized surgical studies whether comparative or non-comparative. The global ideal score is 16 for non-comparative studies and 24 for comparative studies.

### Data Analysis

Meta-analyses were conducted in order to evaluate the association between inflammation indicators and the risk of developing severe COVID-19 (10). For studies that presented continuous data as medians and inter-quartile ranges, the estimate of the means and standard deviations was performed according to the method described by Wan et al. (11). The mean difference (MD/WMD) or the standardized mean difference (SMD) and their related 95% confidence intervals (CIs) were calculated to evaluate the discrepancy of indicators between the non-severe and severe COVID-19 groups. For the pooled analysis of the relationship between indicators and the severity of COVID-19, odds ratio (OR) and related 95% CI were pooled to calculate the effective value.

The Review Manager version 5.3 and STATA software (version 12.0) were used for data analysis. The heterogeneity between studies was assessed by the chi-squared and I-squared tests, with values of 0–25, 25.1–75, and 75.1–100% indicating a low, moderate, and high degree of heterogeneity, respectively. If *I*^2^ > 50%, a random-effect model was used to calculate the effect value. Otherwise, a fixed-effect model was performed. All *P*-values were two-tailed with a significant level at 0.05. Publication bias (number of studies >10) was evaluated using Begg's funnel plots and Begg's rank correlation test, and the significance was set to *P* < 0.05.

## Results

### Literature Information

**Figure 1:**
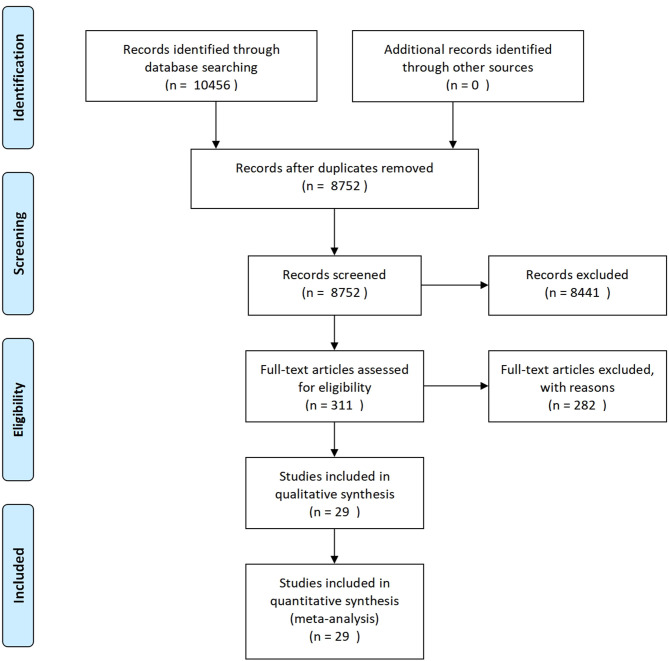
PRISMA flow chart of the study selection procedure.

In the initial search, 10,456 potentially relevant records were found in the PubMed, Web of Science, EMBASE, and CNKI databases (Figure 1). All papers were screened by reading their titles, and 1,704 of them were excluded due to being duplicates found in different databases. After evaluating the abstracts, 8,441 studies were eliminated due to presenting data that were irrelevant to our aim. After carefully reading the full text of the remaining 311 studies, 282 papers that did not meet the inclusion criteria were further excluded. Finally, 29 articles met the inclusion criteria and were included in qualitative synthesis, but some indicators were not described in all articles.

### Characteristics of Included Studies

A total of 29 studies (8, 11–38) published between Feb 2020 and Apr 2020 were identified, and all these studies were retrospective cohort studies of a design with 4,911 patients enrolled in this meta-analysis. The main characteristics of the included studies are summarized and presented in Table 1. Eleven studies were conducted in Wuhan city, the epicenter of COVID-19 outbreak. Only one study was national; it was the largest and included 1,099 COVID-19 patients. The other 17 studies were accomplished in several cities of China outside Wuhan. All patients in selected studies were diagnosis and confirmed as COVID-19. Sample sizes of all studies ranged from 41 to 1,099. The extracted inflammation or immune indicator comprises white blood cell, neutrophil, lymphocyte, monocyte, T cell, helper T cell, cytotoxic T cell, erythrocyte sedimentation rate, Interleukin-6, neutrophil-lymphocyte ratio, C-reactive protein, and procalcitonin. In addition, none of the studies were considered to be seriously flawed according to the MINORS assessment. The 29 included studies scored between 18 and 21.

**Table 1:** Characteristics of included studies in this meta-analysis.

**References**	**Location**	**Sample size (*n*, male)**	**Mean/median age**	**COVID-19 severity**		**Extracted indicators**	**Minors score**
				**Severe**	**Non-severe**		
Bin et al. (12)	Wuhan	54 (30)	53.9	9	45	WBC; L	18
Cai et al. (23)	Shenzhen	298 (145)	47.5	58	240	CRP; PCT; WBC; N; L; ESR; IL-6	21
Chen et al. (33)	Wuhan	150 (84)	59	24	126	CRP	18
Chen et al. (34)	Chongqing	139 (76)	46	31	108	WBC; N; L; NLR	18
Chen et al. (35)	Guangzhou	296 (137)	NA	30	266	WBC; N; L; NLR	20
Fang et al. (36)	Anhui	79 (45)	45.1	24	55	CRP; WBC; N; L	18
Gao et al. (37)	Beijing	90 (43)	53	22	55	CRP; PCT; WBC; L; ESR; IL-6	20
Guan et al. (38)	national	1,099 (637)	47	173	926	WBC; L	18
Hou et al. (39)	Chengdu	56 (29)	48	11	38	L; CD3; CD4; CD8; NLR	20
Huang et al. (13)	Wuhan	41 (30)	49	13	28	PCT; WBC; N; L	21
Li et al. (15)	Wuhan	62 (32)	NA	22	18	CRP; PCT; WBC; N; L; CD3; CD4; CD8	19
Li et al. (14)	Zhuzhou	80 (40)	47.8	17	63	CRP; PCT; WBC; L; ESR	20
Li et al. (16)	Chongqing	83 (44)	45.5	25	58	CRP; PCT; WBC; N; L; M	18
Li et al. (17)	Beijing	46 (21)	45.6	6	40	CRP; PCT; WBC; N; L	18
Liu et al. (18)	Wuhan	78 (39)	38	11	67	CRP; PCT; WBC; N; L; ESR	18
Long et al. (19)	Jingzhou/Xiangyang	301 (150)	51	36	245	NLR	18
Lu et al. (20)	Wuhan	101 (34)	NA	34	67	CRP; WBC; N; L; CD3; CD4; CD8	19
Peng et al. (21)	Wuhan	112 (53)	62	16	96	CRP; PCT; WBC; N; L; M; NLR	19
Qin et al. (8)	Wuhan	452 (235)	58	286	166	CRP; PCT; WBC; N; L; M; CD3; CD4; CD8; ESR; IL-6; NLR	20
Wan et al. (22)	Chongqing	153 (77)	NA	21	132	CRP; PCT; L; CD3; CD4; CD8	21
Wan et al. (24)	Chongqing	135 (72)	47	40	95	CRP; PCT; WBC; N; L	19
Wang et al. (25)	Wuhan	138 (75)	56	36	102	WBC; N; L; M	21
Wu et al. (26)	Wuhan	201 (128)	51	84	117	CRP; WBC; N; L; M; CD3; CD4; CD8; ESR; IL-6	19
Xiang et al. (32)	Jiangxi	49 (33)	42.9	9	40	CRP; PCT; WBC; N; L; CD3; CD4; CD8; ESR	18
Yang et al. (27)	Hangzhou	93 (56)	46.4	24	69	CRP; WBC; N; L; M; NLR	19
Yuan et al. (28)	Chongqing	223 (106)	46.5	31	192	PCT; WBC; L	19
Zhang et al. (29)	Wuhan	138 (71)	57	56	82	CRP; PCT; WBC; L	21
Zhang et al. (30)	Beijing	74 (35)	52.7	9	56	CRP; PCT; WBC; N; L	20
Zheng et al. (31)	Changsha	161 (80)	45	30	131	CRP; WBC; L	18

*WBC, white blood cell; N, neutrophil; L, lymphocyte; M, monocyte; CD3, T cell; CD4, helper T cell; CD8, cytotoxic T cell; ESR, erythrocyte sedimentation rate; IL-6, Interleukin-6; NLR, neutrophil-lymphocyte ratio; CRP, C-reactive protein; PCT, procalcitonin; NA, not available*.

### Immune Cells and Disease Severity in Patients With COVID-19

There is little information about the underlying mechanisms of severe COVID-19 development and further investigations are urgently needed. Qin et al. (8) previously suggested that COVID-19 might damage lymphocytes, especially T lymphocytes, and the immune system was impaired during the period of disease. Also, several studies discovered an increased level of neutrophils along with a decrease in lymphocyte numbers in patients with COVID-19 (13, 16, 29). These findings indicated that neutrophils or lymphocytes could be a potential risk factor for the progression of SARS-CoV-2-infected patients. To test this, we conducted a meta-analysis in order to investigate whether lymphocytes or other immune cells were significantly associated with increased disease severity of COVID-19 (Table 2).

**Table 2:** The association between immune cells and disease severity in patients with COVID-19.

**Immune cells**	**Number of studies**	**Participants**	**Mean difference (95% CI)**	***P***	**Effects model**	**Heterogeneity**	
						***I*^**2**^**	***P*_**h**_**
White blood cell	25	4,278	0.83 (0.41, 1.25)	<0.001	REM	77	<0.001
Neutrophil	18	2,446	1.50 (1.01, 1.98)	<0.001	REM	69	<0.001
Lymhocyte	27	4,480	−0.36 (−0.43, −0.30)	<0.001	REM	73	<0.001
T cell	7	637	−332.48 (−496.93, −168.03)	<0.001	REM	92	<0.001
Helper T cell	7	637	−204.15 (−289.97, −118.33)	<0.001	REM	88	<0.001
Cytotoxic T cell	7	637	−107.23 (−182.78, −31.68)	<0.001	REM	92	<0.001
Monocyte	7	1,128	−0.01 (−0.03, 0.01)	0.28	FEM	0	0.28

*CI, confidence intervals; FEM, fixed-effects model; REM, random-effects model; P_h_, p-value of Q-test for heterogeneity*.

**Figure 2:**
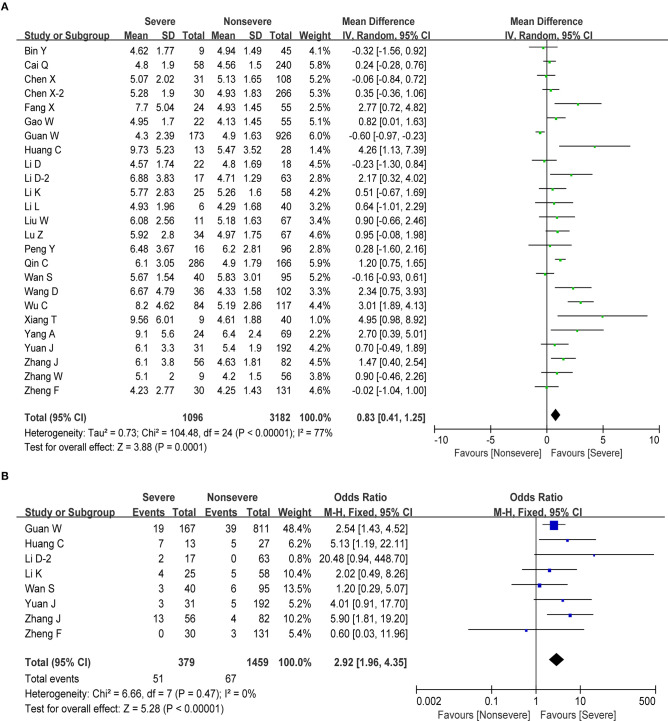
Correlation between WBC and disease severity of COVID-19. **(A)** Forest plot of mean difference in WBC count. **(B)** Forest plot of OR for the association of elevated WBC with disease severity.

Information on white blood cell (WBC) was available in 25 studies, including 4,278 patients with COVID-19. The test showed that these studies have certain heterogeneity (*I*^2^ = 77%, *P* < 0.001), and the random effect model was used. The estimated pooled MD for these studies revealed a significant increase in number of WBC in severe COVID-19 group (MD = 0.83, 95% CI: 0.41–1.25, *P* < 0.001; Figure 2A). In addition, our analysis revealed that, compared to COVID-19 patients with normal levels of WBC, COVID-19 patients who presented with an increased number of WBC (>10 × 10^9^/L) had an ~3-fold higher risk of developing severe disease with a combined odds ratio (OR) of 2.92 (95% CI: 1.96–4.35, *P* < 0.001; Figure 2B). Eight studies were considered to be homogeneous, and the fixed effect model was used (*I*^2^ = 0%, *P* = 0.47). Therefore, we suggest that COVID-19 patients who present with an abnormal WBC count should be carefully monitored and managed according to these results.

Neutrophils are the most abundant white blood cell in the human body, numbering an average of 4,150 cells/μL (50–70% of the total number of WBC). Information on neutrophils was available in 18 studies, including 758 cases in the severe group and 1,688 cases in non-severe group. Since heterogeneity (*I*^2^ = 69%) exceeded 50%, a random effects model was adopted. Similarly, we observed a significant increase in number of neutrophils in severe COVID-19 group (MD = 1.50, 95% CI: 1.01–1.98, *P* < 0.001; Table 2).

**Figure 3:**
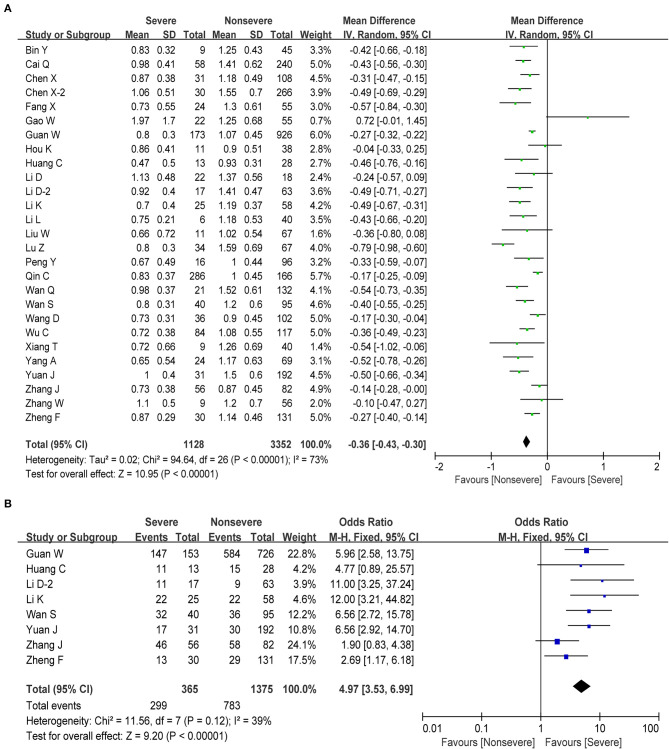
Correlation between lymphocyte and disease severity of COVID-19. **(A)** Forest plot of mean difference in lymphocyte count. **(B)** Forest plot of OR for the association of lymphopenia with disease severity.

Lymphocytes are an important cellular component of the body's immune response function, the main performer of almost all immune functions of the lymphatic system, and a frontline “soldier” to fight external infections and monitor cell mutations in the body. We obtained information about lymphocytes in 27 studies, including 4,480 cases. The pooled analysis revealed that the number of lymphocytes decreased in patients with severe disease compared with non-severe patients (MD = −0.36, 95% CI: from −0.43 to −0.30, *P* < 0.001; Figure 3A). Next, we performed a meta-analysis in order to examine the association between lymphopenia (<1 × 10^9^/L) and disease severity in patients with COVID-19. We found that pronounced lymphopenia was strongly associated with increased disease severity (OR = 4.97, 95% CI: 3.53–6.99, *P* < 0.001; Figure 3B) with moderate heterogeneity (*I*^2^ = 39%, *P* = 0.12). Regarding the changes of several lymphocyte subtypes between severe and non-severe COVID-19 patients, we found that the number of total T cells, helper T cells, and cytotoxic T cells decreased as the disease progresses (Table 2). However, our meta-analysis revealed no significant correlation between monocyte and severe COVID-19 (MD = −0.01, 95% CI: −0.03–0.01, *P* = 0.28; Table 2).

### Neutrophil-Lymphocyte Ratio (NLR) and Disease Progression

**Figure 4:**
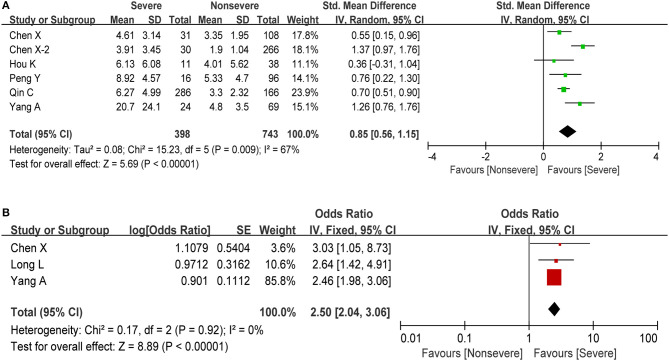
Meta-analysis for NLR in COVID-19 cases. **(A)** Forest plot of mean difference in NLR. **(B)** Forest plot of OR for the correlation of elevated NLR with disease severity.

The neutrophil-lymphocyte ratio (NLR), derived from the absolute neutrophil and absolute lymphocyte counts of a full blood count, is an potential marker of the systemic inflammatory response (40). A rising neutrophil count and a falling lymphocyte count indicate the intensity of the inflammatory response and damage to the immune system, respectively. In this study, we also observed a significant increase in the number of neutrophils and a significant decrease in the lymphocyte count during the severe phase. Therefore, higher NLR could be a potential maker for predicting the disease progression. We obtained information about NLR in six studies including 1,141 cases. The estimated pooled MD for these six studies indicated that severe patients have a higher NLR than non-severe patients (MD = 0.85, 95% CI: 0.56–1.15, *P* < 0.001; Figure 4A). At the same time, elevated NLR was significantly associated with increased disease severity with the pooled OR being 2.50 (95% CI: 2.04–3.06, *P* < 0.001; Figure 4B), demonstrating that elevated NLR could be a predictor of disease progression in COVID-19 patients.

### Inflammation-Related Markers and Disease Severity in COVID-19

**Figure 5:**
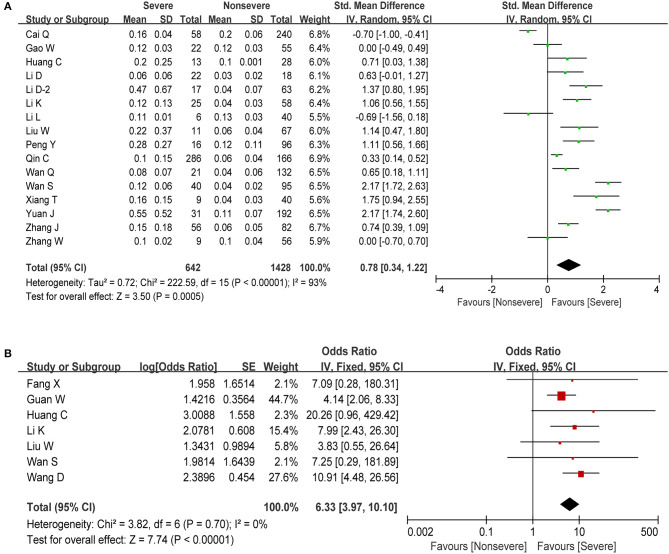
Meta-analysis for PCT in COVID-19 cases. **(A)** Forest plot of comparison of the included studies. **(B)** Forest plot of OR for the correlation of elevated PCT with disease severity.

Procalcitonin (PCT), used as a marker of severe inflammation, is released during infections caused by bacteria, fungi, and parasites but is normal or only slightly elevated in viral infections (41). In this meta-analysis, we obtained information about PCT in 16 studies including 2,070 cases. The estimated pooled MD for these studies revealed a significant increase in PCT (SMD = 0.78, 95% CI: 0.34–1.22, *P* < 0.001; Figure 5A). The pooled analysis showed a high degree of heterogeneity in PCT levels (*I*^2^ = 93%, *P* < 0.001). Then, we performed another meta-analysis in order to examine the putative association between elevated PCT (>0.5 ng/ml) and COVID-19 severity. As shown in Figure 5B, our results revealed that patients who present with elevated PCT have a significantly increased risk of developing severe COVID-19 (OR = 6.33, 95% CI: 3.97–10.10, *P* < 0.001) with low study heterogeneity (*I*^2^ = 0%, *P* = 0.70).

**Figure 6:**
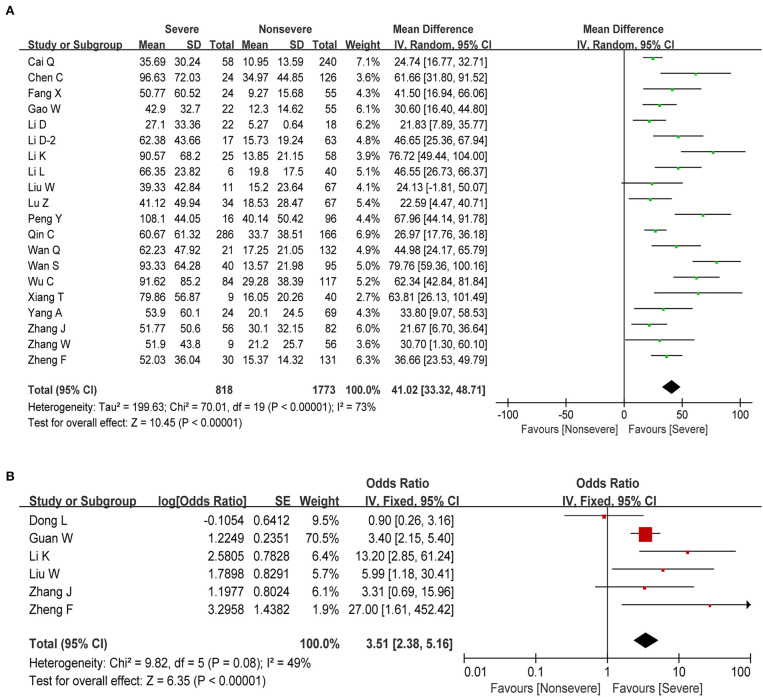
Correlation between CRP and disease severity of COVID-19. **(A)** Forest plot of mean difference in CRP. **(B)** Forest plot of OR for the association of increased CRP with disease severity.

C-reactive protein (CRP) is an acute-phase response protein synthesized by the liver and elevated in response to inflammatory diseases. It plays a vital role in protection against infection, prevention of autoimmunity, and regulation of the inflammatory response. Information on CRP was available in 20 studies, including 2,591 patients with COVID-19. The estimated pooled MD indicated that severe patients have a higher level of CRP than non-severe patients (MD = 41.02, 95% CI: 33.32–48.71, *P* < 0.001; Figure 6A), with moderate study heterogeneity (*I*^2^ = 73%). Moreover, we found that elevated CRP (above normal range) was significantly correlated with increased disease severity in COVID-19 (OR = 3.51, 95% CI: 2.38–5.16, *P* < 0.001; Figure 6B) with moderate heterogeneity (*I*^2^ = 49%, *P* = 0.08). Regarding other major Inflammation-related markers such as erythrocyte sedimentation rate (ESR) and Interleukin-6 (IL-6), we found that ESR and IL-6 were both significantly associated with increased disease severity (Table 3).

**Table 3:** The association between inflammation-related indicators and disease severity in patients with COVID-19.

**Inflammation-related indicators**	**Number of studies**	**Participants**	**Mean difference/Std. mean difference (95% CI)**	***P***	**Effects model**	**Heterogeneity**	
						***I*^**2**^**	***P*_**h**_**
Procalcitonin	16	2,070	0.78 (0.34, 1.22)	<0.001	REM	93	<0.001
C-reactive protein	20	2,591	41.02 (33.32, 48.71)	<0.001	REM	73	<0.001
Erythrocyte sedimentation rate	5	706	18.37 (6.59, 30.15)	0.002	REM	84	<0.001
Interleukin-6	3	951	0.72 (0.13, 1.30)	0.02	REM	94	<0.001
Neutrophil-lymphocyte ratio	6	1,141	0.85 (0.56, 1.15)	<0.001	REM	67	0.009

*CI, confidence intervals; REM, random-effects model; P_h_, p-value of Q-test for heterogeneity*.

### Publication Bias

**Figure 7:**
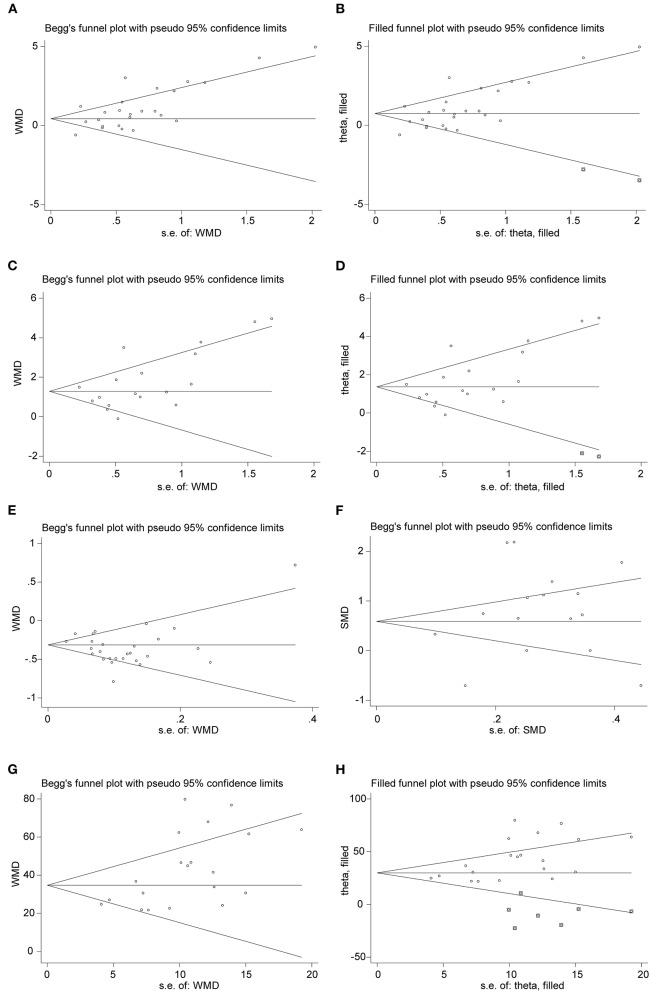
Funnel plots of comparison of the included studies. **(A,B)** Funnel plot and adjusted funnel plot of mean difference in WBC count. **(C,D)** Funnel plot and adjusted funnel plot of mean difference in neutrophil count. **(E)** Funnel plot of mean difference in lymphocyte count. **(F)** Funnel plot of Std. mean difference in PCT. **(G,H)** Funnel plot and adjusted funnel plot of mean difference in CRP.

Publication bias was originally defined as the publication or non-publication of studies depending on the direction and statistical significance of the results. Publication bias was examined by the funnel plot and Begg's rank correlation test. As shown in Figure 7, distribution of the funnel plot was nearly symmetric and no evidence of publication bias in lymphocyte (*P* = 0.967) and PCT (*P* = 0.964). However, it was asymmetric in the meta-analyses of WBC, neutrophil, and CRP. Therefore, the trim and fill method was adopted to adjust publication bias (42). After adjustment, distribution of the funnel plot was more symmetric than before (Figure 7). At the same time, we found that the adjusted pooled MD did not change significantly, which indicated that the publication bias had little impact on the analysis and the analysis results were relatively stable (Figures S1–S3).

## Discussion

The majority of COVID-19 patients have relatively mild symptoms, but a considerable number of patients progress to severe pneumonia and even eventually develop acute respiratory distress syndrome (ARDS), septic shock, and/or multiple organ failure (2). Therefore, it is of great significance to study the immunological characteristics of peripheral blood in severe patients for timely diagnosis, precise treatment, delaying or halting the progression of the disease, and reducing mortality.

As is well-known, viral infection is closely related to the human immune system; good immune function can help the body eliminate foreign microorganisms, control infection, and eventually restore health (43). Dysregulation of immune cell responses and consequently immunologic abnormality are thought to play important roles in the severity of virus-induced disease (44). Indeed, previous studies of novel coronavirus infection have suggested that the severe and aberrant host immune response are responsible for the severity of COVID-19 (16, 24). At the same time, peripheral blood immune-inflammatory parameters will also change significantly with the progression of COVID-19 disease. Therefore, emerging researches were focus on these accessible laboratory data for assessing and predicting clinical severity in patients with COVID-19. One of the most prominent factors related to the severity and outcomes of the Middle East respiratory syndrome coronavirus (MERS-CoV) disease is the hematological change in the white blood cell population (45). Also, several studies have addressed the difference of baseline leukocyte counts between the clinical stages in COVID-19 patients. Mo et al. (46) reported that refractory patients had higher level of neutrophils in comparison with general patients. In patients with severe COVID-19, but not in patients with mild disease, lymphopenia is a common feature, with drastically reduced numbers of CD4+T cells, CD8+ T cells, B cells, and natural killer (NK) cells (2, 8, 13). Qin et al. (8) investigated 452 patients with laboratory confirmed COVID-19 and found that severe cases were likely to have higher neutrophil count but lower lymphocyte count compared with non-severe patients; the NLR thus tended to be higher in the severe group. Long et al. (19) reported that neutrophil-lymphocyte ratio ≥2.973 (HR = 2.64, 95% CI: 1.42–4.91, *P* = 0.002) was an independent risk factor for progression of COVID-19 by multivariate Cox regression analyses. Moreover, some studies showed that higher levels of inflammatory cytokines, chemokines, and NLR were correlated with the severity of the disease (13, 47). Zhu et al. (48) demonstrated that high level of IL-6 and CRP were independent risk factors for assessing the severity of COVID-19.

This meta-analysis was conducted to assess the association between immune-inflammatory parameters and increased disease severity in COVID-19 patients. In the present study, we firstly utilized the available data from 25 included studies with a total of 4,278 patients to obtain the pooled results to evaluate the difference in WBC count between a severe and non-severe group. We found that the white blood cell counts of severe patients tended to be higher than that of less severe patients. Next, the effect of WBC count on the risk of developing severe COVID-19 was further examined. The pooled results statistically supported the conclusions that elevated WBC count was strongly associated with the deterioration of disease in COVID-19 patients (OR = 2.92, 95% CI: 1.96–4.35, *P* < 0.001). Similarly, the number of neutrophils was found to be higher in severe COVID-19 (MD = 1.50, 95% CI: 1.01–1.98, *P* < 0.001). However, patients with serious disease tend to have a reduced total lymphocyte count as well as virous lymphocyte subtypes count compared to non-severe COVID-19 patients. In addition, COVID-19 patients who presented with lymphopenia had an ~5-fold higher risk of developing severe disease (OR = 4.97, 95% CI: 3.53–6.99, *P* < 0.001). Consistent with previous reports, this meta-analysis also indicated that the incidence of high NLR had significant association with illness severity (MD = 0.85, 95% CI: 0.56–1.15, *P* < 0.001) and elevated NLR could act as a predictor for exacerbation of COVID-19 (OR = 2.50, 95% CI: 2.04–3.06, *P* < 0.001). Moreover, other common immune-inflammatory parameters, such as PCT and CRP, could also predict the deterioration of disease (PCT: OR = 6.33, 95% CI: 3.97–10.10, *P* < 0.001; CRP: OR = 3.51, 95% CI: 2.38–5.16, *P* < 0.001). In this study, ESR and IL-6 were also found to be correlated with increased disease severity in COVID-19 patients.

SARS-CoV-2 infection can activate innate and adaptive immune responses. A rapid and well-coordinated innate immune response is the first line of defense against viral infections, while uncontrolled inflammatory innate responses and impaired adaptive immune responses may lead to harmful tissue damage, both locally and systemically (49). Taking both the levels of neutrophil and lymphocyte into account, NLR may be a better biomarker for systemic inflammation and illness severity than single neutrophil or lymphocyte count. In this study, we confirmed that an increase in NLR usually indicated higher disease severity with more clinical evidence. The following reasons may explain the finding. On one hand, most patients with severe COVID-19 exhibit substantially elevated serum levels of proinflammatory cytokines. Meanwhile, neutrophils can be triggered by virus-related inflammatory factors produced by lymphocyte and endothelial cells (such as interleukin-6 and interleukin-8, tumor necrosis factor-alpha and granulocyte colony stimulating factor, and interferon-gamma) (50). The triggered neutrophils produce reactive oxygen species (ROS) and other cytotoxic mediators, which may dampen the virus infection (51). Moreover, neutrophils are able to release neutrophil extracellular traps (NETs), which are a sticky web of DNA conjugated with antimicrobial enzymes (such as myeloperoxidase and histones), resulting in the capture and the killing of different pathogens, including viruses (52–54). On the other hand, lymphocytes did not show a significant decrease in the early stage of viral infection but significantly decreased in severe and critically ill patients (34). The main reasons for the decrease or exhaustion of lymphocyte in severe cases may be the following: viruses attack target cells and directly damage cells; viral infection causes immune cells to enter an activated state and participate in the anti-viral process, resulting in severe damage and apoptosis; systematic inflammation stimulates the production of neutrophil and speed up the apoptosis of lymphocyte; severe COVID-19 patients tend to present an increase of PCT and CRP, indicating a potential bacterial co-infection; and bacterial co-infection or superinfection might affect the immune response.

Although the sample size of this study is large (4,911 COVID-19 cases from 29 clinical studies enrolled), some limitations should be noted meanwhile. Firstly, the primary research design of the studies included in this study were retrospective cohorts with insufficient demonstration ability, limiting their ability to infer definitive causality. Secondly, all included original clinical cohort studies were conducted in China, which limits the ability of this study to extrapolate other patient populations in other countries. Thirdly, the high statistic heterogeneity could be found in calculating pooled MD, which may relate to large variation among studies in the sample size.

In summary, we came to the cautious conclusion that immune-inflammatory parameters such as WBC, lymphocyte, NLR, PCT, and CRP were correlated with disease severity and could be used as potentially important risk factors for disease progression. In addition, increased NLR levels reflecting an enhanced inflammatory process may also suggest a poor prognosis. Therefore, surveillance of immune-inflammatory parameters, especially NLR, may be helpful in the diagnosis, early screening and predicting of severe illness, and treatment of COVID-19.

## Data Availability Statement

All datasets generated for this study are included in the article/Supplementary Material.

## Author Contributions

XF, BC, MX, and GC conceived and designed the study. XF, SL, and QS selected the studies, collected the data, and drafted and revised the paper. XF and JZ analyzed data. All authors interpreted the results, read, and approved the final version of the manuscript.

## Conflict of Interest

The authors declare that the research was conducted in the absence of any commercial or financial relationships that could be construed as a potential conflict of interest.
