# Supplementary material for: Immune-Inflammatory Parameters in COVID-19 Cases: A Systematic Review and Meta-Analysis
Source: Front Med (Lausanne). 2020 Jun 9;7:301. doi: 10.3389/fmed.2020.00301 (PMC7295898; doi:10.3389/fmed.2020.00301)
Supplement: Supplementary file 1 [file Data_Sheet_1.DOC]

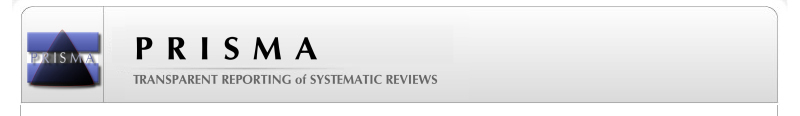
**PRISMA 2009 Flow Diagram**

**Screening**

**Included**

**Eligibility**

**Identification**

Records identified through database searching
(n = 10456 )

Additional records identified through other sources
(n = 0 )

Records after duplicates removed
(n = 8752 )

Records screened
(n = 8752 )

Records excluded
(n = 8441 )

Full-text articles assessed for eligibility
(n = 311 )

Full-text articles excluded, with reasons
(n = 282 )

Studies included in qualitative synthesis
(n = 29 )

Studies included in quantitative synthesis (meta-analysis)
(n = 29 )
