# Supplementary material for: Immune-Inflammatory Parameters in COVID-19 Cases: A Systematic Review and Meta-Analysis
Source: Front Med (Lausanne). 2020 Jun 9;7:301. doi: 10.3389/fmed.2020.00301 (PMC7295898; doi:10.3389/fmed.2020.00301)
Supplement: Supplementary file 3 [file Data_Sheet_3.DOCX]

Figure S1. Trim and fill method for WBC meta-analysis.

Figure S2. Trim and fill method for neutrophil meta-analysis.

Figure S3. Trim and fill method for CRP meta-analysis.
