# Supplementary material for: Immune-Inflammatory Parameters in COVID-19 Cases: A Systematic Review and Meta-Analysis
Source: Front Med (Lausanne). 2020 Jun 9;7:301. doi: 10.3389/fmed.2020.00301 (PMC7295898; doi:10.3389/fmed.2020.00301)
Supplement: Supplementary file 4 [file Data_Sheet_4.DOC]

Search strategy

PubMed

| No. | Query |
| --- | --- |
| #1 | Search: (((((COVID-19[Title/Abstract]) OR (Novel Coronavirus-Infected Pneumonia[Title/Abstract])) OR (2019 novel coronavirus[Title/Abstract])) OR (NCP[Title/Abstract])) OR (2019-nCoV[Title/Abstract])) OR (SARS-CoV-2[Title/Abstract]) |

Web of Science

| No. | Query |
| --- | --- |
| #1 | TS=(COVID-19) OR TS=(Novel Coronavirus-Infected Pneumonia) OR TS=(2019 novel coronavirus) OR TS=(NCP) OR TS=(2019-nCoV) OR TS=(SARS-CoV-2) |

EMBASE

| No. | Query |
| --- | --- |
| #1 | 'covid 19':ab,ti OR 'novel coronavirus-infected pneumonia':ab,ti OR '2019 novel coronavirus':ab,ti OR ncp:ab,ti OR '2019 ncov':ab,ti OR 'sars cov 2':ab,ti |

CNKI

| No. | Query |
| --- | --- |
| #1 | ( ( ( ( ( (Subject=COVID-19 OR Title=COVID-19) OR (Subject=Novel Coronavirus-Infected Pneumonia OR Title=Novel Coronavirus-Infected Pneumonia) ) OR (Subject=2019 novel coronavirus OR Title=2019 novel coronavirus) ) OR (Subject=NCP OR Title=NCP) ) OR (Subject=2019-nCoV OR Title=2019-nCoV) ) OR (Subject=SARS-CoV-2 OR Title=SARS-CoV-2) ) |
